# A six-year descriptive analysis of hospitalisations for ambulatory care sensitive conditions among people born in refugee-source countries

**DOI:** 10.1186/1478-7954-5-9

**Published:** 2007-10-03

**Authors:** Ignacio Correa-Velez, Zahid Ansari, Vijaya Sundararajan, Kaye Brown, Sandra M Gifford

**Affiliations:** 1 Refugee Health Research Centre, La Trobe University, Victoria 3086, Australia; 2 Chronic Disease Surveillance and Epidemiology Section, Public Health Branch, Department of Human Services, 15/50 Lonsdale Street, Melbourne, Victoria 3000, Australia; 3 Programs Branch, Health Surveillance and Evaluation Section, Department of Human Services, 19/50 Lonsdale Street, Melbourne, Victoria 3000, Australia; 4 Statewide Elective Surgery Program, Access and Metropolitan Performance Branch, Department of Human Services, 18/50 Lonsdale Street, Melbourne, Victoria 3000, Australia

## Abstract

**Background:**

Hospitalisation for ambulatory care sensitive conditions (ACSHs) has become a recognised tool to measure access to primary care. Timely and effective outpatient care is highly relevant to refugee populations given the past exposure to torture and trauma, and poor access to adequate health care in their countries of origin and during flight. Little is known about ACSHs among resettled refugee populations. With the aim of examining the hypothesis that people from refugee backgrounds have higher ACSHs than people born in the country of hospitalisation, this study analysed a six-year state-wide hospital discharge dataset to estimate ACSH rates for residents born in refugee-source countries and compared them with the Australia-born population.

**Methods:**

Hospital discharge data between 1 July 1998 and 30 June 2004 from the Victorian Admitted Episodes Dataset were used to assess ACSH rates among residents born in eight refugee-source countries, and compare them with the Australia-born average. Rate ratios and 95% confidence levels were used to illustrate these comparisons. Four categories of ambulatory care sensitive conditions were measured: total, acute, chronic and vaccine-preventable. Country of birth was used as a proxy indicator of refugee status.

**Results:**

When compared with the Australia-born population, hospitalisations for total and acute ambulatory care sensitive conditions were lower among refugee-born persons over the six-year period. Chronic and vaccine-preventable ACSHs were largely similar between the two population groups.

**Conclusion:**

Contrary to our hypothesis, preventable hospitalisation rates among people born in refugee-source countries were no higher than Australia-born population averages. More research is needed to elucidate whether low rates of preventable hospitalisation indicate better health status, appropriate health habits, timely and effective care-seeking behaviour and outpatient care, or overall low levels of health care-seeking due to other more pressing needs during the initial period of resettlement. It is important to unpack dimensions of health status and health care access in refugee populations through ad-hoc surveys as the refugee population is not a homogenous group despite sharing a common experience of forced displacement and violence-related trauma.

## Background

Hospitalisation for ambulatory care sensitive conditions (ACSHs) has become a recognised tool to measure access to primary care [1-3]. Ambulatory Care Sensitive Conditions (ACSCs) have been defined as those conditions for which "the provision of timely and effective outpatient care can help to reduce the risks of hospitalization by either preventing the onset of an illness or condition, controlling an acute episodic illness or condition, or managing a chronic disease or condition" [4](p.163). Some examples of ACSCs are influenza, pneumonia, asthma, hypertension, congestive heart failure, diabetes complications, and nutritional deficiencies.

The rate of ACSHs has been found to be influenced by a number of factors such as income [1,5], health insurance status [6], access to regular primary care [7], continuity of care [8], and race and ethnicity [3]. Minority race/ethnic groups have been observed to be at greater risk of being hospitalised for a preventable condition [3,9-11] and their higher hospitalisation rates persist after adjusting for disease prevalence [9].

Given the challenges of the growing problem of forced displacement, and in view of the internationalisation of refugee resettlement [12], it is important for governments, public health professionals, and health service providers in resettlement countries to obtain evidence on humanitarian arrivals' access to primary care and preventable hospitalisation. Timely and effective outpatient care is highly relevant to refugee populations given the past exposure to torture and trauma, and poor access to adequate health care in their countries of origin and during flight [13,14]. ACSHs among refugee populations remain largely undocumented. With the aim of examining the hypothesis that people from refugee backgrounds have higher ACSHs than people born in the country of hospitalisation, this study analysed a six-year state-wide hospital discharge dataset to estimate ACSH rates for residents born in refugee-source countries and compared them with the Australia-born population. Australia has a publicly-funded universal health care scheme for all citizens and permanent residents, including humanitarian entrants, which provides access to free treatment in public hospitals, and free or subsidised treatment by general practitioners, specialists and optometrists.

## Methods

### Data overview

We used hospital discharge data between 1 July 1998 and 30 June 2004 from the Victorian Admitted Episodes Dataset (VAED). The VAED contains data on all admitted patients from public and private acute hospitals in the state of Victoria. It also includes admitted patient activity submitted by acute facilities in rehabilitation and extended care institutions and day procedure centres [15]. Although VAED is an administrative dataset that is collected for operational reasons, it provides useful information about acute health care use and health-related outcomes. Clinical data are stored as ICD-10-AM [16] codes in 25 (from 1998/99 to 2002/03) to 40 (2003/04) diagnosis and procedures fields. We obtained permission to use de-identified data from the Victorian Department of Human Services.

### Study population

In this study, country of birth was used as a proxy indicator of refugee status. The VAED lacks data on immigration status and on ethnicity beyond country of birth (with the exemption of Indigenous Australians). Refugee-source countries are defined as those countries where large numbers of people have been forcibly displaced due to persecution, armed conflict and war [13]. In this study, we have included eight countries for which the majority of entrants to Victoria have arrived under the immigration humanitarian program [17,18]. These countries are: Afghanistan, Bosnia-Herzegovina, Burma, Eritrea, Ethiopia, Iraq, Somalia and Sudan.

### Definition of Ambulatory Care Sensitive Conditions (ACSCs)

We used the classification of ACSC applied in the Victorian Ambulatory Care Sensitive Conditions Study [19], which draws from previous research [6,20]. According to this classification, there are three categories of ACSCs:

(a) Acute ACSCs (reducing morbidity and pain through timely and appropriate treatment): This category includes acute disease for which hospitalisation is avoidable, for example, dehydration/gastroenteritis, kidney infection, perforated ulcer, cellulitis, pelvic inflammatory disease, ear, nose and throat infections, and dental conditions. In this category, the conditions may not be preventable but theoretically do not result in hospitalisation if adequate and timely primary care is received.

(b) Chronic ACSCs (reducing the effect of chronic disease and prolonging life): This category includes selected chronic diseases for which hospitalisation is avoidable, for example, diabetes, asthma, angina, hypertension, congestive heart failure, chronic obstructive pulmonary disease. In this category, the conditions may be preventable through behaviour modification and lifestyle change, but they can also be managed effectively through primary care to prevent deterioration and hospitalisation.

(c) Vaccine-preventable ACSCs (reducing the incidence of preventable diseases): This includes hospitalisation for influenza, bacterial pneumonia, tetanus, measles, mumps, rubella, pertussis and polio-conditions for which vaccination is available. In this category, the actual conditions are deemed to be preventable, rather than the hospitalisation. There is, however, a misclassification bias with respect to vaccine-preventable ACSC in the context of this study. Admissions due to vaccine-preventable ACSC cannot be assessed among children born in Australia to refugee families because they are classified as Australia-born.

The ACSCs identified using the ICD-10-AM codes in the diagnosis fields of the VAED are shown in Table 1.

**Table 1:** Ambulatory Care Sensitive Conditions (ACSC) and ICD-10-AM codes used in the analyses

**Category**	**ICD-10-AM codes**	**Notes**
Influenza and pneumonia	J10 J11 J13 J14 J153 J154 J157 J159 J168 J181 J188	In any diagnosis field, excludes cases with secondary diagnosis of D57, and people under 2 months
Other vaccine preventable	A35 A36 A37 A80 B05 B06 B161 B169 B180 B181 B26 G000 M014	In any diagnosis field
Asthma	J45 J46	Principal diagnosis only
Congestive heart failure	I50 I110 J81	Principal diagnosis only
Diabetes complications	E101 to E108, E110 to E118, E130 to E138, E140 to E148	In any diagnosis field
Chronic obstructive pulmonary disease	J20 J41 J42 J43 J44 J47	Principal diagnosis only, J20 only with diag2 of J41 J42 J43 J44 J47
Angina	I20 I240 I248 I249	Principal diagnosis only, excludes cases with procedures codes NOT in blocks 1820 to 2140
Iron deficiency anaemia	D501 D508 D509	Principal diagnosis only
Hypertension	I10 I119	Principal diagnosis only
Nutritional deficiencies	E40 to E43, E550 E643	Principal diagnosis only
Dehydration and gastroenteritis	E86 K522 K528 K529	Principal diagnosis only
Pyelonephritis	N10 N11 N12 N136 N390	Principal diagnosis only
Perforated/bleeding ulcer	K250 K251 K252 K254 K255 K256 K260 K261 K262 K264 K265 K266 K270 K271 K272 K274 K275 K276 K280 K281 K282 K284 K285 K286	Principal diagnosis only
Cellulitis	L03 L04 L08 L88 L980 L983	Principal diagnosis only, excludes cases with any procedure except those in blocks 1820 to 2016 of if procedure is 30216-02 30676-00 30223-02 30064-00 34527-01 34527-00 90661-00 and this is the only listed procedure
Pelvic inflammatory disease	N70 N73 N74	Principal diagnosis only
Ear, nose and throat infections	H66 H67 J02 J03 J06 J312	Principal diagnosis only
Dental conditions	K02 to K06, K08 K098 K099 K12 K13	Principal diagnosis only
Convulsions and epilepsy	O15 G40 G41 R56	Principal diagnosis only
Gangrene	R02	In any diagnosis field

### ACSC admission rates and rate ratios

ACSC admission rates were age-standardised using the direct method [21]. Annual Victorian population estimates for most of the refugee-source countries included in this analysis were not readily available. Therefore, population figures for individual refugee-source countries were estimated by adding the number of arrivals in Victoria born in that country [17] to the number of individuals recorded at the previous census [22]. The Australia-born population was estimated using figures from the 1996 and 2001 censuses [22]. The estimated resident population of Victoria was used as the standard population.

A random sample of 100,000 Australia-born admissions was taken for each year. Rate ratios are used throughout the paper to compare the ACSC between Australia-born and refugee-source country-born [21]. A rate ratio greater than 1.0 indicates that ACSC admissions were higher for refugee-source-country-born than for Australia-born persons. Confidence levels of 95% are used to assess and report the precision of ACSC admission rates and rate ratios. We used the method based on the gamma distribution [23] to calculate confidence intervals for age-adjusted rates.

## Results

The number and standardised ratios of total, acute, chronic, and vaccine-preventable ACSHs for persons born in refugee-source countries over the six-year period (1998/99 and 2003/04) is shown in Table 2.

**Table 2:** Number of hospital admissions (N), standardised ratios (SR) and 95% confidence levels (95%CI) for total, acute, chronic, and vaccine-preventable ambulatory care sensitive conditions (ACSCs) for persons born in refugee-source countries, 1998/99 to 2003/04

**ACSCs**	**1998/99**		**1999/2000**		**2000/01**		**2001/02**		**2002/03**		**2003/04**		**Total N**
		
	N	SR (95% CI)	N	SR (95% CI)	N	SR (95% CI)	N	SR (95% CI)	N	SR (95% CI)	N	SR (95% CI)	
Total	254	18.7 (16.1,21.7)	298	21.4 (18.6, 24.5)	518	29.8 (27.0,32.8)	572	32.4 (29.5,35.6)	569	31.9 (29.0,35.0)	680	35.5 (32.5, 38.7)	2891
Acute	112	6.4 (5.1, 8.1)	145	7.8 (6.4, 9.6)	199	8.1 (6.9, 9.5)	259	11.7 (10.1,13.4)	249	10.5 (9.0, 12.1)	322	13.0 (11.4, 14.8)	1286
Chronic	124	11.4 (9.3, 14.0)	138	12.5 (10.3,15.2)	307	21.2 (18.8,23.9)	296	20.1 (17.7,22.7)	311	21.1 (18.7,23.8)	343	22.2 (19.7, 25.0)	1519
Vaccine-preventable	18	0.8 (0.5, 1.6)	17	1.2 (0.7, 2.2)	15	0.7 (0.4, 1.4)	24	1.1 (0.6, 1.7)	18	0.9 (0.5, 1.5)	25	1.0 (0.6, 1.6)	117

### Total ambulatory care sensitive conditions

**Figure 1:**
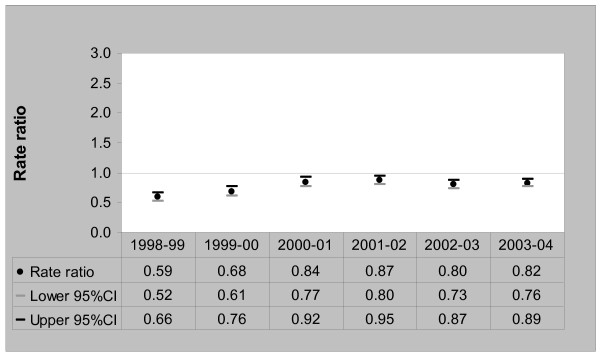
Total ACSCs Admission Rate Ratios – Refugee-source country-born (Australia-born = 1), 1998/99 to 2003/04.

Between 1998 and 2004, standardised rates of admissions for total ACSCs doubled among the refugee-source country-born, from 18.7 per 1000 persons [95%CI, 16.1–21.7] in 1998/99 to 35.5 per 1000 persons [95%CI, 32.5–38.7] in 2003/04. Among the Australia-born population, the rates of total ACSCs admissions also increased from 31.9 per 1000 persons [95%CI, 31.3–32.6] in 1998/99 to 43.2 per 1000 persons [95%CI, 42.4–44.1] in 2003/04. Figure 1 compares total ACSCs admission rate ratios between refugee-source country-born and the Australia-born average.

### Acute ambulatory care sensitive conditions

**Figure 2:**
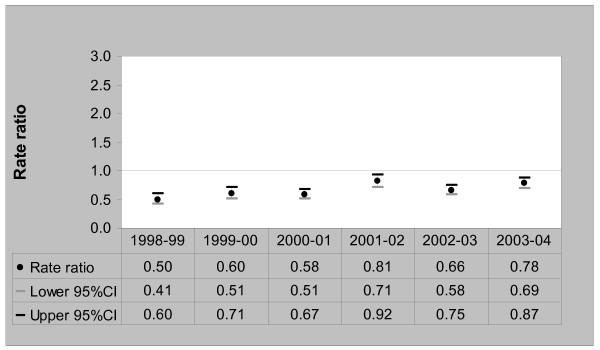
Acute ACSCs Admission Rate Ratios – Refugee-source country-born (Australia-born = 1), 1998/99 to 2003/04.

For the refugee-source country-born, rates of acute ACSC admissions doubled between 1998/99 and 2003/04, increasing from 6.4 per 1000 persons [95%CI, 5.1–8.1] in 1998/99 to 16.7 per 1000 persons [95%CI, 11.4–14.8] in 2003/04. Among the Australia-born, admission rates of acute ACSCs also increased from 12.9 per 1000 persons [95%CI, 12.5–13.3] in 1998/99 to 16.7 per 1000 persons [95%CI, 16.2–17.3] in 2003/04. A comparison of acute ACSC admission rate ratios between the two population groups is presented in Figure 2.

### Chronic ambulatory care sensitive conditions

**Figure 3:**
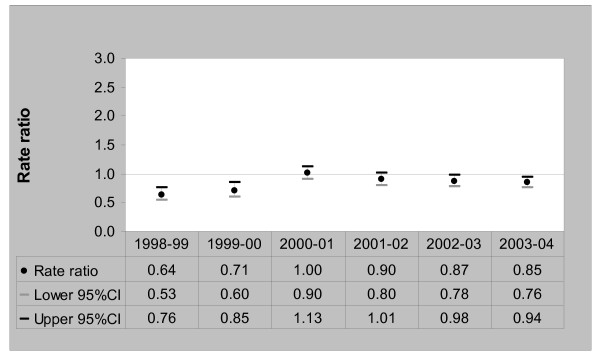
Chronic ACSCs Admission Rate Ratios – Refugee-source country-born (Australia-born = 1), 1998/99 to 2003/04.

The standardised rates of admission for chronic ACSCs doubled among the refugee-source country-born between 1998/99 and 2003/04. Rates increased from 11.4 admissions per 1000 persons [95%CI, 9.3–14.0] in 1998/99 to 22.2 per 1000 persons [95%CI, 19.7–25.0] in 2003/04. Rates of admissions amongst the Australia-born increased from 17.9 per 1000 persons [95%CI, 17.4–18.5] in 1998/99 to 26.2 per 1000 persons [95%CI, 25.5–27.0] in 2003/04. Figure 3 compares chronic ACSC admission rate ratios between the refugee-source country-born and the Australia-born over the six-year period.

### Vaccine-preventable ambulatory care sensitive conditions

**Figure 4:**
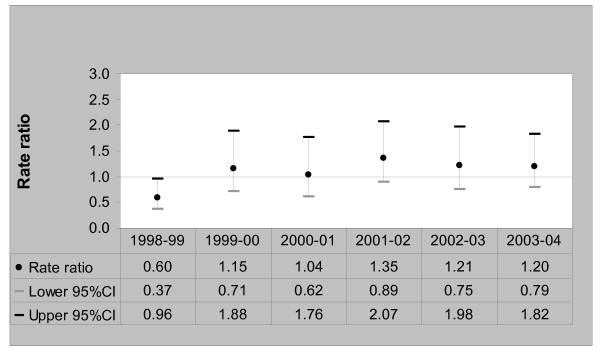
Vaccine-preventable ACSCs Admission Rate Ratios – Refugee-source country-born (Australia-born = 1), 1998/99 to 2003/04.

There was no consistent pattern of admission rates for vaccine-preventable ACSCs among the refugee-source country-born population over the six-year period. The lowest admission rate was 0.7 per 1000 persons [95%CI, 0.4–1.4] in 2000/01 and the highest was 1.2 per 1000 persons [95%CI, 0.7–2.2] in 1999/2000. Among the Australia-born, the rates of vaccine-preventable ACSC admissions decreased from 1.4 per 1000 persons [95%CI, 1.3–1.6] in 1998/99 to 0.8 per 1000 persons [95%CI, 0.7–1.0] in 2003/04. A comparison of vaccine-preventable ACSC admission rate ratios between the two groups is shown in Figure 4.

## Discussion

To our knowledge, this is the first study that has investigated ACSHs in resettled refugee populations. Contrary to our hypothesis, the study has found lower total and acute rates and similar chronic and vaccine-preventable rates of ACSHs among refugee-source country-born persons when compared to the Australia-born population.

A number of studies have previously examined the effects of race and ethnicity on ACSHs in industrialised countries. Shah et al [24] found that the Aboriginal population in Ontario, Canada, had significantly higher admission rates for ACSCs compared with the general Ontario population. Similarly, Stamp et al [11] reported higher ACSHs among Australian Aboriginal and Torres Strait Islander populations than non-Aboriginal and Torres Strait Islanders. Other studies have consistently found higher preventable hospitalisation rates among African Americans and Hispanics than non-Hispanic whites in the USA [3,9,10].

The effects of immigration status on ACSHs have been scarcely investigated. Glazier et al [25] examined ACSHs in high recent-immigration areas in Toronto, Canada, and found that the areas with the highest quintile of recent immigration reported the highest relative rates of ACSHs. In contrast, a study by DeLia [26] used births to immigrant mothers as a proxy for immigrant population in the USA and found this variable to be associated with lower ACSHs. These studies however did not discriminate across immigrant categories.

Given the lack of international literature on ACSHs among refugee communities it is not possible to determine whether the findings of our study are unique to Australia or may apply to other resettlement countries. Although refugees may share some of the characteristics of other immigrants, they have experienced extraordinary circumstances of stress and trauma prior to entering the country of resettlement, and therefore are likely to have poorer health status, particular vulnerability to specific health conditions, and higher need to health care [14,27]. In this context, we had hypothesised that refugee populations would have higher rates of hospital admissions, including ACSHs, than the native-born. On the contrary, we have previously found either similar or lower rates of hospital utilisation among residents born in refugee-source countries compared with the Australia-born population [28]. Our findings demonstrate also either similar or lower rates of ACSHs among the refugee-source country-born population.

Following Ansari et al categorisation [2], there are at least three groups of factors that may provide an explanation for these findings: health factors, social determinants, and behavioural risk factors. The health factors group include disease prevalence, propensity to seek care, and physician supply. Our results may indicate that recent refugee arrivals have lower morbidity, as measured by ACSHs, thus confirming the 'healthy migrant effect'[14]. The health requirement that refugees undergo before entering Australia may be selecting those who are healthier [29]There may be also a time lag in the built up of relevant risk factors for some ACSCs such as asthma, diabetes, hypertension and ischaemic heart disease [13] as new arrivals are exposed to the same environment and adopt some of the unhealthy habits (i.e. smoking, drinking, eating junk food) of the receiving society [14]. The overall increase of ACSHs among the refugee-source country-born towards Australia-born averages over the six-year time period may support this argument. The findings may also suggest that refugees seek care earlier in the course of chronic disease and therefore are less likely to require hospitalisation. It might be also that Australia's universal health care cover and the adequate levels of physician supply available in urban areas, where most refugees settle, lead to 'timely and effective outpatient care' hence reducing the risks of preventable hospitalisations. Nevertheless, there is recent evidence of important health issues in primary care settings (i.e. inadequate vaccinations, nutritional deficiencies, infectious/parasitic diseases, dental disease, musculoskeletal problems) among newly arrived refugees in Australia and of the need for comprehensive health assessments for this population [30,31]. While State governments in Australia are implementing a number of strategies to address the health care needs of recently arrived refugee communities [32], there is emerging evidence of the multiple barriers that affect access to primary and acute health care among this population [30,33,34]. Among these barriers are language barriers, financial disadvantage, lack of information about health issues, poor understanding of how to access health care facilities, and structural barriers within these services [33]. Free or subsidised medical care, like the one provided in a number of refugee resettlement countries, "does not imply equal access to services nor equity in service" [35] (p.165).

In the social determinants group, appropriate levels of income, employment, and education may benefit health care-seeking behaviour or patient education, which may lead to low ACSHs [4,36]. However, although people from refugee backgrounds are not a homogeneous population, there is evidence of economic hardship, high levels of unemployment [37], and low levels of educational attainment [17,38] among recent refugee arrivals to Australia. When considering the behavioural risk factors group, our findings may suggest good health habits (i.e. low levels of smoking and alcohol consumption, adequate nutrition and physical activity) among people born in refugee-source countries, which may lead to a decreased risk of ACSHs. Although there is emerging evidence of nutritional deficiencies among recent refugee arrivals [30], mostly due to poor nutrition while living in refugee camps prior to resettling in Australia, and some indication of refugees being at an increased risk for substance misuse [39], very little is known about health habits among recent refugee arrivals to developed countries.

Our study has several limitations. First, the lack of baseline information on immigration status for people attending acute health care settings prompted the use of country of birth as a proxy for refugee status. Our estimates indicate that approximately 80% of arrivals between 1996 and 2004 born in the eight refugee-source countries entered Australia under the humanitarian resettlement program [28]. Second, as data reporting disease prevalence by country of birth was not readily available for the selected refugee-source countries, we were unable to adjust for this variable. This is important as "differing disease prevalence might account for differences in preventable hospitalization" [9] (p.249). However, previous research has found that, independent of prevalence, better access to health care in Victoria was associated with lower ACSH rates [2]. Third, we were unable to adjust for length of time in Australia as it was not available in the hospital dataset. It can be argued that recent refugee arrivals would be less likely to seek health care during the initial period of resettlement as they are focused in addressing 'more pressing' issues such as housing, employment, children education, and family reunion. Those who have been in the country for a shorter period of time may also have less knowledge about how to navigate through the health care system. Fourth, the analyses could not be extended to smaller areas such as the local governments and primary care partnerships due to small numbers. Small area analyses on refugee populations along with ad-hoc surveys will be useful in identifying access barriers refugees face in health care settings. These access barriers can be economic, structural, geographic, or cultural. Unpacking these dimensions of access will have the potential to provide the basis for policy makers to develop targeted public health and health services interventions.

## Conclusion

This study represents a significant step towards the development of an evidence-based knowledge around resettled refugee populations and preventable hospitalisation. Despite emerging evidence of important health issues among recently arrived refugee populations, we have found that preventable hospitalisation rates among people born in refugee-source countries are either similar or lower than Australia-born averages. Our study has been unable to elucidate whether low rates of preventable hospitalisation among this refugee population indicate better health status, appropriate health habits, timely and effective care-seeking behaviour and outpatient care, or overall low levels of health care-seeking due to other more pressing needs during the initial period of resettlement. More research is needed to clarify these issues. It is important to unpack dimensions of health status and health care access in refugee populations through ad-hoc surveys as the refugee population is not a homogenous group despite sharing a common experience of forced displacement and violence-related trauma. Factors such as ethnicity, gender, culture, socio-economic and educational status, health systems and services in the country of origin, and the particular contexts of displacement, flight, and resettlement play an important role in a refugee's experience of ill health and, consequently, of health care utilisation and health-related outcomes in countries of resettlement.

## Abbreviations

ACSCs – Ambulatory Care Sensitive Conditions

ACSH – Hospitalisation for Ambulatory Care Sensitive Conditions

ICD-10-AM – International Statistical Classification of Diseases and Related Health Problems, 10th Revision, Australian Modification

VAED – Victorian Admitted Episodes Dataset

## Competing interests

The author(s) declare that they have no competing interests.

## Authors' contributions

IC-V participated in the design of the study, conducted the statistical analysis, and drafted the manuscript.

ZA provided advice on the conceptualisation and measurement of ACSCs, design of the study and data analysis, and reviewed the manuscript.

VS participated in the study design, provided advice on data analysis, and reviewed the manuscript.

KB participated in the design of the study and reviewed the manuscript.

SMG participated in the design of the study and reviewed the manuscript.
